# Companion diagnostics and clinical drug development in oncology: a survey based on US FDA data

**DOI:** 10.3389/fmed.2026.1938709

**Published:** 2026-10-30

**Authors:** Jan Trøst Jørgensen

**Affiliations:** Medical Science, Dx-Rx Institute, Fredensborg, Denmark

**Keywords:** accelerated approval, breakthrough therapy, companion diagnostics, drug-diagnostic codevelopment, fda, oncology, orphan drug, study design

## Abstract

Over the past few decades, the drug-diagnostic codevelopment model has gradually transformed oncology drug development. In this model, a predictive biomarker assay, known as a companion diagnostic (CDx), is developed alongside the drug. The incorporation of CDx assays in clinical drug development has facilitated the use of the enrichment trial design, in which patients are selected based on molecular characteristics linked to the drug mechanism of action. In this survey, oncology drugs associated with a CDx assay approved by the FDA until the end of 2025 were investigated, and drug class, clinical trial design, study endpoint, patient population, Orphan Drug status, and expedited review were recorded. During this period, 87 cancer drugs were identified, of which 62 (71%) were indicated for treatment of solid tumors. Kinase inhibitors were the dominant drug class, accounting for 34 drugs (55%). A large proportion of the drugs had obtained Orphan Drug status (63%) and Breakthrough Therapy designation (77%). The average patient population sufficient to obtain FDA approval was 279, with 16 (26%) drugs approved based on fewer than 100 patients, often supported by single-arm, open-label clinical trials. For 49 (79%) of the drugs, efficacy documentation was generated based on one single clinical trial. The primary endpoint in these trials was most often objective response rate and progression-free survival. When evaluating the enrichment trial design, the number of patients required to demonstrate clinical efficacy and obtain FDA approval is low compared to the traditional all-comers design, which means that safety evaluation could be compromised.

## 1 Introduction

For decades, randomized double-blind clinical trials have been the scientific standard for evaluating the therapeutic efficacy and safety of drugs in humans. However, since the turn of the century, the drug-diagnostic codevelopment model has gradually changed this picture, particularly with respect to the clinical evaluation of drugs aimed at treating patients with oncological and hematological malignancies. Recent advances in tumor biology and molecular pathophysiology have enabled the development of a number of target-specific drugs. These drugs are effective only in subsets of patients with specific molecular characteristics (1, 2). To identify these patients, different predictive biomarker assays have been developed to select the appropriate patient population likely to respond (3). These assays are termed companion diagnostics (CDx), which the Food and Drug Administration (FDA) and other regulatory bodies define as an *in vitro* diagnostic device that provides information that is essential for the safe and effective use of a corresponding therapeutic product (4, 5). For the precise selection of patients for treatment, it is crucial that these CDx assays undergo analytical and clinical validation before being introduced in the clinic, which must be done in a timely manner to be available simultaneously with the drugs they are meant to guide (2).

The drug-diagnostic codevelopment model was first successfully introduced when trastuzumab was developed for the treatment of patients with metastatic breast cancer whose tumors overexpressed the HER2 protein in the 1990s (6). An immunohistochemical (IHC) assay to identify tumor HER2 expression was developed concurrently with the drug to select the population of likely responding patients (7). Since the simultaneous approval of trastuzumab and its IHC assay in 1998, the number of cancer drugs associated with a CDx assay has increased to more than 80 (8). Clinical trials performed to document the safety and efficacy of these targeted drugs have used different versions of the enrichment trial design. To investigate the clinical trial designs for these drugs and the implications with respect to study endpoints, patient population, orphan drug (OD) status, and expedited development and review a survey was conducted using FDA publicly available data.

## 2 Methodes

The oncological drugs and biologics associated with CDx assays were identified based on the FDA “List of Cleared or Approved Companion Diagnostic Devices (*in vitro* and Imaging Tools)” for the period from 1998 to the end of 2025 (8). For the identified drugs and biologics, efficacy documentation leading to their approval was assessed based on data available from “Drugs@FDA” (9). Data pertaining to the drug class and study design elements, such as blinding, number of patients, endpoints, and efficacy outcomes were collected. Data on the initial approval of the drugs were collected, or if the drug was associated with a CDx at a later time point. The OD status was retrieved from the FDA database of “Orphan Drug Designations and Approvals” (10). Furthermore, information on expedited development and review, such as Priority Review, Breakthrough Therapy, Accelerated Approval, and Fast Track, was collected from the regulatory history in the New Drug Application (NDA) or Biologics License Application (BLA) for the individual drugs as available at “Drugs@FDA” (9, 11). With respect to Breakthrough Therapy designations, supplemental data were retrieved from the Friends of Cancer Research database (12). Descriptive data analyses were performed using Excel.

## 3 Results

At the end of 2025, the total number of regulatory-approved drugs associated with CDx assays was 87. Of these 87 drugs, 62 (71%) were used for the treatment of different types of solid tumors, and the remaining 25 were primarily used for the treatment of hematological malignancies. Among the 62 oncological drugs, 34 (55%) were classified as kinase inhibitors, followed by monoclonal antibodies and small-molecule inhibitors, accounting for 12 (19%) and 10 (16%), respectively. The remaining six drugs (10%) were antibody-drug conjugates or bispecific antibodies (8, 9).

For the 62 oncological drugs associated with a CDx assay, the mean number of patients included in the clinical efficacy documentation was 279. This figure does not include the patients included in the large retrospective analysis of the different panitumumab trials with respect to *KRAS* mutations. The drug with the lowest number of patients with respect to the evaluation of efficacy was dordaviprone, a small-molecule inhibitor, which in 2025 was approved for the treatment of diffuse midline glioma patients harboring an *H3 K27M* mutation (13). In contrast, the monoclonal antibody zolbetuximab had the highest number of patients with respect to clinical efficacy documentation. Zolbetuximab in combination with chemotherapy was in 2024 approved for treatment of patients with metastatic HER2-negative gastric or gastroesophageal junction adenocarcinoma whose tumors expressed the claudin-18.2 protein (14). The approval of zolbetuximab was based on clinical efficacy data from 1,072 patients enrolled in two double-blind randomized trials. Notably, for 16 of the 62 oncological drugs developed together with a CDx assay, the number of patients needed to document their efficacy and obtain FDA approval was less than 100. In Table 1. these drugs are listed together with information on biomarkers, indications, OD status and expedited development and review.

**Table 1:** For 16 of the 62 oncological drugs developed with a CDx assay, the number of patients needed to document their efficacy and obtain FDA approval was less than 100. The drugs are listed are listed together with information on biomarkers, indications, OD status and expedited development and review (9). .

Drugs	Biomarker	Indication	No. of pt.	Orphan Drug	Priority Review	Fast Track	Accelerated Approval	Breakthrough Therapy
Dordaviprone	*H3 K27M*	Glioma	50	Yes	Yes	Yes	Yes	Yes
Entrectinib	*NTRK1, NTRK2, NTRK3*	Solid tumors	54	Yes	Yes	No	Yes	Yes
Larotrectinib	*NTRK1, NTRK2, NTRK3*	Solid tumors	55	Yes	Yes	No	Yes	Yes
Avutometinib plus defactinib	*KRAS*	Ovarian cancer	57	Yes	Yes	No	Yes	Yes
Pembrolizumab	PD-L1—TPS ≥ 50%	NSCLC	61	No	No	No	Yes	Yes
Zanidatamab	HER2	Biliary tract cancer	62	Yes	No	Yes	Yes	Yes
Sevabertinib	*HER2*	NSCLC	70	Yes	Yes	No	Yes	Yes
Dostarlimab	dMMR proteins	Endometrial Carcinoma	71	No	No	No	Yes	Yes
Tovorafenib	*BRAF*	Glioma	76	Yes	Yes	No	Yes	Yes
Avapritinib	*PDGFRA*	GIST	81	Yes	No	Yes	Yes	Yes
Amivantamab	*EGFR*	NSCLC	81	No	No	No	Yes	Yes
Telisotuzumab vedotin	c-Met protein	NSCLC	84	No	No	No	Yes	Yes
Sunvozertinib	*EGFR*	NSCLC	85	No	Yes	No	Yes	Yes
Erdafitinib	*FGFR3*	Urothelial Cancer	87	No	Yes	No	Yes	Yes
Durvalumab	dMMR proteins	Endometrial Carcinoma	95	No	Yes	No	No	Yes
Capmatinib	*MET*	NSCLC	97	Yes	Yes	No	Yes	Yes

NSCLC, Non-Small Cell Lung Cancer; GIST, GastroIntestinal Stromal Tumor.

Investigating the study design for all 62 drugs associated with a CDx assay, the single-arm open-label design dominated. As shown in Table 2, this design was used for more than half of the drugs (52%), followed by the randomized trial design, which was used in relation to 27 drugs (44%). For three drugs (4%), both single-arm and randomized trials were used to document their clinical efficacy. These drugs include the kinase inhibitor vemurafenib and the monoclonal antibodies trastuzumab and cetuximab (9). Regarding blinding, 53 (85%) drugs used an open-label design, and a double-blind design was used for the remaining nine (15%) drugs.

**Table 2:** The study design for drugs associated with a CDx assay to document their clinical efficacy, which led to their FDA approval (9).

Study Design	No. of drugs	%
Single arm	32	52
Randomized	27	44
Both single arm and randomized	3	4
Blinding		
Open label	53	85
Double blind	9	15

Efficacy documentation was generated based on one single trial for 49 (79%) of the 62 drugs. Two trials were required to evaluate the clinical efficacy of 10 drugs (16%), and three (5%) drugs required three or more trials. The primary efficacy endpoint was the objective response rate (ORR) for 37 drugs (60%), and progression-free survival (PFS) and overall survival (OS) were the primary endpoints for 25 drugs (40%).

For all 62 drugs, 39 (63%) had obtained OD status (10). With regard to expedited development and review, Breakthrough Therapy designation was granted for 48 drugs (77%). Similar figures for Accelerated Approval, Fast Track, and Priority Review were 35 (56%), 15 (24%), and 39 (63%), respectively (9, 12). With regard to expedited development and review, a large proportion of the drugs obtained several designations. For example, dordaviprone was granted Accelerated Approval, Fast Track, Priority Review, and Breakthrough Therapy as shown in Table 1.

## 4 Discussion

Over the past few decades, we have observed a shift in cancer drug development, moving away from non-specific drugs towards molecular targeted drugs. This has also impacted the size of the targeted populations for these drugs, as the use of molecular stratification by CDx assays clearly impacts the real number of patients benefiting from such treatment. An example of this is the drug dordaviprone, which in 2018 was granted OD designation and was regulatory approved by the FDA in August 2025 for the treatment of patients with diffuse midline glioma harboring an *H3 K27M* mutation (13). This approval was based on data from an efficacy population of 50 patients enrolled in five small, open-label, and single-arm clinical trials. *H3 K27M* mutant diffuse midline glioma is a rare type of brain tumor with an incidence of 0.06 per 100,000 people, corresponding to approximately 2,100 patients diagnosed per year in the USA, which substantiates the small number of patients available for clinical development (15).

This change from non-specific drugs to molecular targeted drugs is also reflected in the number of OD designations. Nearly two-thirds (63%) of all oncological drugs associated with a CDx assay approved by the FDA in the period from 1998 to the end of 2025 have obtained an OD status (10). When discussing the number of patients included in the clinical evaluation of targeted cancer drugs, it is important to have the size of the patient populations in mind, as described for the development of dordaviprone. To obtain an OD status in the US, the drug must not be indicated for the treatment of diseases that affect more than 200,000 individuals, or the development of the drug will not be profitable within seven years following FDA approval (10). As shown in Table 1, for several drugs with an OD status, their approval was based on clinical data from less than 100 patients in single-arm clinical trials (9). In defense of the limited amount of clinical data and the use of the single-arm trial design, it is important to consider that these drugs are indicated for the treatment of small patient populations, where it would be difficult to perform a large randomized clinical trial. Furthermore, with respect to the non-randomized single-arm trial design, which was used for more than half of the drugs, it must be remembered that for most of these new targeted therapies, relevant comparator drugs did not exist at the time of their development. In general, when discussing OD status, oncology has since the OD Act was passed in 1983 account for 38% all designations and approvals (16). This is explained by the increasing molecular subdivision of cancers diseases using different type of molecular analytical technologies such as next-genaration sequencing, polymerase chain reaction and IHC.

The possibility of obtaining regulatory approval for a targeted cancer drug based on a single-arm clinical study with a limited number of patients has been debated. However, it is important to have in mind that this approach has positively contributed to the progress of cancer treatment in recent years and has provided patients with access to more effective treatments early on. Furthermore, it must be remembered that many of these targeted drugs address an unmet medical need, which remains substantial in the treatment of cancer. Although efficacy can be demonstrated relatively efficiently in this type of clinical trial, the downside is the safety evaluation. It is challenging to obtain even a rough estimate of the safety profile of a new drug in such small patient populations, especially when it comes to rarer events. It is important to have sufficient information on the safety profile of a new drug when treatment is initiated, and the enrichment trial design often lags behind with respect to this critical issue. Consequently, safety information must be obtained in the post-approval phase, which is suboptimal. Overall, the compromise here is fast access for the patient to a new and more effective treatment at the cost of safety, which, in most situations, is ethically justifiable. When discussing the treatment of life-threatening diseases, such as cancer, regulatory authorities in the USA, Europe, and other countries appear to share a common opinion by prioritizing efficacy and rapid access to more effective treatments for patients over safety by approving these drugs despite limited safety data.

With respect to the 62 targeted drugs associated with a CDx assay, more than half of them (56%) were granted Accelerated Approval, which allows earlier approval of drugs for the treatment of serious conditions that fill an unmet medical need based on a surrogate endpoint (17). In this survey, the use of surrogate endpoints, such as ORR, was used in the clinical trials for 37 drugs (60%). However, this does not exempt pharmaceutical or biotech companies from verifying the clinical benefit of the drugs. For oncological drugs, this often means overall survival and further safety data, which are stipulated as postmarketing requirements by the FDA.

With respect to the other element of the FDA expedited development and review program, Fast Track and Priority Review, a relatively large proportion of 62 targeted drugs were qualified for this. With regard to Fast Track, 15 drugs (24%) were assigned this status, which is a process introduced to facilitate the development and expedite the review of drugs to treat serious conditions and fill an unmet medical need (18). With respect to Priority Review, this means that the goals of the FDA are to take action on an application within 6 months, compared to 10 months under standard review. This designation is used for drugs that could significantly improve the safety or effectiveness of the treatment of serious conditions (19). Thirty-nine (63%) of the targeted drugs associated with a CDx assay were granted a Priority Review designation, thereby obtaining a shorter review time.

The novelty of targeted oncological drugs has also been recognized by the FDA, as Breakthrough Therapy designation was granted for 48 (77%) of the 62 drugs associated with a CDx assay. Breakthrough Therapy designation has been introduced to expedite the development and review of drugs that are intended to treat a serious condition and where preliminary clinical evidence indicates that the drug may demonstrate substantial improvement over available therapy (20). The targeted oncological drugs interfere with specific molecular targets that are critical for cancer cell growth, survival, and dissemination. In recent decades, there has been a shift in both the types of drugs developed and the methodologies used in their development, particularly concerning the assessment of their clinical efficacy. The specificity of targeted drugs calls for different clinical trial designs compared to past drugs, such as chemotherapeutics, where large randomized phase III trials, often including several hundred patients required to document their benefit (21, 22). The ability of CDx assays to select likely responding patients increases the power of individual clinical trials, which has impacted both trial design and the number of patients required to document efficacy. In fact, for 16 (26%) oncology drugs associated with a CDx assay, clinical evaluation was performed in a patient population of less than 100. For these drugs, clinical efficacy was frequently evaluated based on a single-arm trial, which subsequently led to FDA approval. The primary endpoint in these trials was most often ORR, matching the initial indication for most of these drugs, which was frequently the treatment of late-stage metastatic diseases (9).

Historically, the drug-diagnostic codevelopment model was introduced in relation to the development of the monoclonal antibody trastuzumab. Concurrently with the development of trastuzumab, an IHC assay was developed and used for patient selection during clinical development (7). After the regulatory approval of trastuzumab for the treatment of breast cancer in 1998, the importance of the enrichment clinical trial design for its development was discussed (23). In relation to this discussion, it was stated that if an HER2 IHC assay had not existed, trastuzumab might have been discarded during clinical development because of insufficient activity in an unselected patient population (24). Despite this statement being aimed at trastuzumab more than 25 years ago, it could also be applied in relation to most of the more than 80 targeted cancer drugs that have subsequently been developed, which also used the drug-diagnostic codevelopment model. These drugs have noticeably improved the treatment of many cancer patients, which also have been recognized by the FDA with respect to the frequents used of the different elements in their expedited development and review program, especially with regards to Breakthrough Therapy designation. In 2025, 16 new small-molecule drugs and biologics were approved for the treatment of cancer, with the majority developed using a CDx assay (25). Over the past few decades, the enrichment trial design has significantly impacted oncology drug development in terms of clinical and regulatory aspects and will likely continue to do so in the future.
